# 147. Defining the Optimal Serial Testing Interval and Features for Identifying Patients with Early SARS-CoV-2 Infection

**DOI:** 10.1093/ofid/ofab466.147

**Published:** 2021-12-04

**Authors:** Sanjat Kanjilal

**Affiliations:** Harvard Medical School and Harvard Pilgrim Healthcare Institute, Jamaica Plain, MA

## Abstract

**Background:**

Serial testing for SARS-CoV-2 is necessary to prevent spread from patients early in infection. Testing intervals are largely derived from viral kinetic studies performed early in the COVID-19 pandemic. Laboratory and epidemiologic data accrued over the past year present an opportunity to use empiric models to define optimal serial testing intervals and features predictive of early infection.

**Methods:**

Retrospective analysis of 15,314 inpatients within the Mass General Brigham healthcare system who had two tests within a 36-hour period between May 1 2020 and May 29 2021. Early infection was defined as having a negative test followed by a positive test. Patients with prior positive tests were excluded. The primary outcome was the proportion of patients in early infection over the total number tested serially, stratified by 4-hour testing intervals from the timestamp of the first test. Multivariate modeling was used to identify features predictive of early infection. Covariates included demographics, body site, PCR assay, location, community incidence, percent positivity, and median / skew of Ct value distributions.

**Results:**

Of 19,971 test pairs, 193 (0.97%) were characterized as a negative followed by a positive within 36 hours. Bivariate analysis showed a close association between negative to positive test pairs during the first surge in spring 2020 that was not present during the winter surge. Negative to positive test pairs were most common in the 12 to 16 hour time interval (51/193, 26%, Figure 1). After controlling for covariates, the Roche cobas assay was more likely to identify patients with a negative to positive test pair relative to the Cepheid Xpert, Hologic Panther Fusion and Roche Liat assays. A second specimen from the lower respiratory tract was more likely to lead to a positive relative to other body sites. Community incidence and Ct value distributions were not predictive and there were no differences between nasal and nasopharyngeal swabs. All 4-hour time intervals from 16 to 36 hours were significant for predicting a negative to positive test pair (Table 1).

Figure 1. Distribution of negative to positive test pairs by 4 hour time intervals

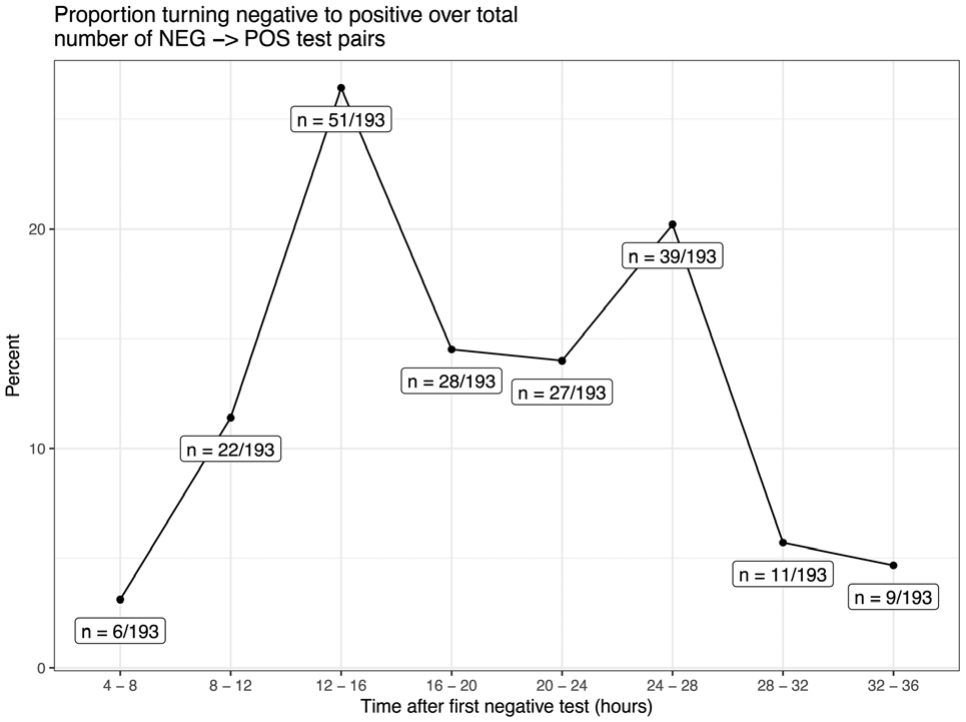

Table 1. Multivariate regression predicting a negative to positive test pair

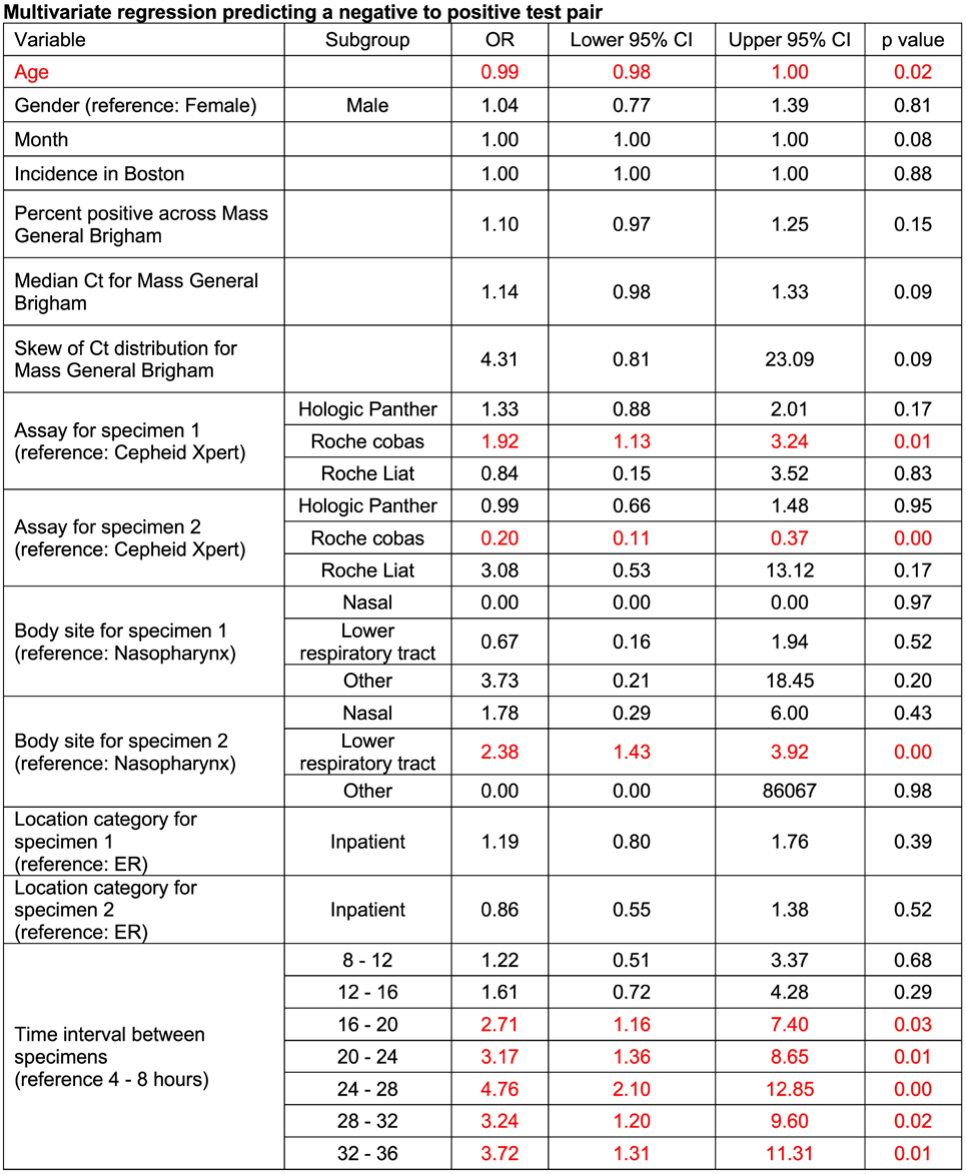

**Conclusion:**

The likelihood of detecting early infection is dependent on PCR platform and body site of sampling. A range of time intervals between 16 to 36 hours after the initial test were likely to identify positive cases.

**Disclosures:**

**Sanjat Kanjilal, MD, MPH**, **GlaskoSmithKline** (Advisor or Review Panel member)

